# Effect of arbuscular mycorrhiza and rhizobium on physiology and yield of peanut under drought conditions

**DOI:** 10.3389/fpls.2024.1468636

**Published:** 2024-11-26

**Authors:** Chorkaew Aninbon, Pattrarat Teamkao, Kiattisak Buram, Tipawan Kaewnoo, Ruttanachira Ruttanaprasert, Anon Janket, Yi Yi Mon, Phissanu Kaewtaphan

**Affiliations:** ^1^ Faculty of Agricultural Technology, King Mongkut’s Institute of Technology Ladkrabang, Bangkok, Thailand; ^2^ Soil Science Research Group, Agricultural Production Sciences Research and Development Division, Department of Agriculture, The Ministry of Agriculture and Cooperatives, Bangkok, Thailand; ^3^ Department of Plant Science, Textile and Design, Faculty of Agriculture and Technology, Rajamangala University of Technology Isan, Surin, Thailand; ^4^ Department of Agronomy, Faculty of Agriculture, Ubon Ratchathani University, Ubon Ratchathani, Thailand; ^5^ Department of Plant Pathology, Yezin Agricultural University, Nay Pyi Taw, Myanmar

**Keywords:** drought tolerance crop, groundnut, microorganism, phenols, AMF

## Abstract

Drought is the one primary issue limiting peanut growth and productivity. The study aimed to investigate the effects of arbuscular mycorrhizal fungi (AMF), rhizobium (Rhi), and their combinations on phenolic content, proline content, growth, and yield of peanut under different soil water regimes. The pot experiments were carried out for two growing seasons under greenhouse conditions and designed based on a 2×3 factorial in randomized complete block design (RCBD) with four replications. Factor A comprised two soil water regimes: field capacity (FC) and 1/3 available soil water (1/3 AW), whereas factor B included three different types of microorganisms: (i) uninoculated control, (ii) arbuscular mycorrhiza (AMF), and (iii) a combination of AMF and rhizobium (Rhi) inoculations. Data were collected for growth, proline content, phenolic content, yield, and yield components. Drought stress significantly reduced in relative water content, leaf area, biomass, yield, and yield components of peanut, whereas leaf phenolic content was increased under drought stress. Higher pod dry weight was achieved under FC conditions (28.87 g plant^-1^), and it was reduced to 16.06 g plant^-1^ under 1/3 FC. Interestingly, AMF+Rhi synergistically increased the leaf area compared with non-incubated peanut under 1/3 FC conditions. AMF-inoculated peanut tended to increase biomass, while the combination of AMF+Rhi tended to have higher yield components compared with uninoculated control, especially for the weight of 100 seeds.

## 1 Introduction

Peanut is mainly grown in tropics and semiarid tropics where drought is a major problem because of unpredictable rainfall and rain distribution (Rachaputia et al., 2021). Crop growth and productivity can be severely affected by drought, which is one of the most important abiotic stresses (Fathi and Tari, 2016). The peanut is typically grown in two main seasons: rainy season and dry season (Sukharomana and Dobkuntod, 2003).

In Thailand, peanut is grown under three production systems, namely, intercropping of peanut with other tree crops and field crops, growing as a major crop in the wet season, and growing after harvest of field crops in the late rainy season without irrigation or in the dry season with irrigation (Sukharomana and Dobkuntod, 2003). Peanut planted in the late rainy season without irrigation and in the dry season with irrigation can experience drought stress because of depletion of stored soil water and insufficient of irrigation water. As a major crop, peanut can be affected by drought due to unpredictable rainfall and distribution. Mid-season drought (Junjittakarn et al., 2016) and terminal drought (Girdthai et al., 2010; Koolachart et al., 2013; Aninbon et al., 2019) are particularly detrimental to pod yield of peanut because the drought events occur during reproductive phases.

Symbiotic nitrogen fixation of leguminous species by association with rhizobium is adopted widely in agriculture to reduce the excessive use of chemical nitrogen fertilizer (Abd-Alla et al., 2023). Peanut with rhizobium inoculation fixed 27.19 kg N ha^−1^. The Rhizobia inoculation increased the amount of nitrogen fixation up to 46% in North Eastern Nigeria (Yakubu et al., 2010), 42%–51% in Petrolina, and 43%–60% in Sergipe, Brazil (Jovino et al., 2022). According to Toomsan et al. (1995), peanut fixed nitrogen in the range of 150 kg N ha^−1^–200 kg N ha^−1^. When the stover was returned to the soil, the net contributions of N from N fixation ranged from 13 kg N ha^−1^–100 kg N ha^−1^. Therefore, peanut contributes to soil improvement, when plant parts of the peanut are added to the soil after harvesting.

The rhizobium directly supports the host plant by nitrogen fixation process including increased solubilization of nutrients via organic acid and siderophore production, and hormone production such as IAA and gibberellin. The Rhizobium also indirectly support the plant by production of antibiotics, catalase, and siderophores that control root pathogens (Jaiswal et al., 2021). The inoculation of Rhizobium isolated from cowpea increased the grain yield of peanut IAC505 (Guimaraes et al., 2019). Rhizobium application with 100% nitrogen recommended dose significantly improved peanut yield, yield-related traits, seed quality, and maximum uptake of major nutrients (nitrogen, phosphorus, and potassium) (Mondal et al., 2020). Moreover, *Rhizobium leguminosarum* could help to reduce drought effects in the two faba bean genotypes (Amine-Khodja et al., 2022).

Arbuscular mycorrhizal fungi (AMF) are now recognized as beneficial microorganisms that enhance plant growth and also reduce drought conditions by producing and extending the hyphae into dry soil and increasing surface contact for improved water absorption by host plants. Additionally, AMF enhance nutrient uptake and morphological adaptations and also influence physiological processes of host plants (Cheng et al., 2021). AMF-inoculated soybeans increase the relative water content (RWC) and photosynthetic rate compared with uninoculated plants, leading to higher dry weight of plants and seeds (Aliasgharzad et al., 2006; Oliveira et al., 2022). Inoculation peanut with AMF also enhances catalase and peroxidase activities in seeds (Sheteiwy et al., 2021). Similarly, inoculation of chickpea with AMF under drought conditions also improves the RWC, stomatal conductance, and photosynthetic rate compared with uninoculated plants (Hashem et al., 2018). Proline can help water absorption from the soil through the osmotic adjustment under drought conditions. Phenols also play an important role in regulating developmental mechanisms and tolerance to various stresses including drought stress. In addition, proline and phenolic contents in wheat also increase under drought stress, and this increase is more prominent in non-AMF inoculated plants than in AMF-inoculated plants. However, phenolic compounds do not show a significant difference between the inoculation methods of AMF.

Most of the studies so far have focused on individual inoculation of AMF and rhizobium (Rhi) on peanut crop, and information on co-inoculation of AMF and rhizobium is still limited. A recent greenhouse study indicated that combined inoculation of AMF and rhizobium on soybean favored the growth response in association with higher P and N uptake (Dobo, 2022). Similar results were also reported in *Phaseolus vulgaris* planted in low-fertility tropical soil (Razakatiana et al., 2020). It is a challenge to explore the co-inoculation of AMF and rhizobium on peanut, particularly under drought conditions. The hypothesis of this study was that application of AMF and/or co-inoculation of AMF and rhizobium would improve peanut proline content, phenolic content, growth, and yield under drought conditions. The aim of this study was to evaluate the effects of AMF, rhizobium (Rhi), and their combinations on phenolic content, proline content, growth, and yield of peanut under two different soil water regimes. The results of this study will provide information on the use of AMF and rhizobium for improving growth and yield of peanut under drought stress.

## 2 Materials and methods

### 2.1 Experimental description

A pot experiment was conducted in a greenhouse at the faculty of Agricultural Technology, King Mongkut’s Institute of Technology Ladkrabang (13.7299° N, 100.7782° E, 0.5 m above sea level) for two growing seasons such as dry season (February–May 2023) and rainy season (August–November 2023). A 2×3 factorial experiment was arranged in a randomized complete block design (RCBD) with four replications. Six pots were used for each replication as subunits. Factor A comprised two different soil water regimes: field capacity (FC) and 1/3 available soil water (1/3 AW). Factor B included three inoculations of arbuscular mycorrhiza (AMF): (i) uninoculated control, (ii) inoculation with AMF, and (iii) a combination of AMF and rhizobium (Rhi). A peanut variety, KKU9, which is a newly released variety in Thailand and is recognized by its high pod yield and good plant architecture, was used in this study. The soil used in this study was Chakraja soil series, which is characterized as loamy sand. The chemical and physical properties of the soil are described in **Table 1**. The field capacity of the soil (FC) was 11.24%, and the permanent wilting point (PWP) was 5.27%.

**Table 1:** The properties of the soil used in this experiment.

Parameters	Mean	S.D.	Meaning
pH 1:1	7.69	0.113	Slightly high
EC 1:5 (mS/cm)	0.04	<0.01	Not saline
OM (%)	0.8748	0.024	Low
Available P (ppm)	29.46	2.47	High
Exchangeable K (ppm)	32.25	1.598	Low
Exchangeable Ca (ppm)	433.06	7.551	Low
Exchangeable Mg (ppm)	57.9	0.551	High
Exchangeable Na (ppm)	16.82	1.258	Low
Extractable Fe (ppm)	31.37	1.06	High
Extractable Mn (ppm)	28.54	0.975	High
Extractable Cu (ppm)	0.365	0.007	Low
Extractable Zn (ppm)	2.27	0.014	Medium

### 2.2 Pot preparation, inoculation, and crop management

The soil was air-dried and sieved through a 4-mm sieve to remove debris and homogenize the soil sample. Thirty six kg of the dry soil was evenly divided and filled into pots in two soil layers in each pot to ensure a uniform bulk density (1.56 g cm^−3^). Water was supplied to all the pots to obtain the soil moisture content at the field capacity level (11.24%).

The Rhi and AMF inoculants were commercial biofertilizers (Rhizobium: *Bradyrhizobium* sp. with 10^6^ CFU g^−1^ and AMF: *Glomus* spp. with 25 spore g^−1^). The soil inoculation was done by putting the inoculum in a hole dug in each pot. The amount of inoculum, 10 g of AMF for AMF-alone treatment, and 10 g each of AMF and Rhizobium for combination were used. Healthy peanut seedlings at 15 days old were transplanted to the center of each pot (1 plant pot^−1^), and water was immediately applied to ensure good establishment.

Manual weeding was done whenever necessary, and compound fertilizer of N-P_2_O_5_-K_2_O (15-15-15) at the rate of 156.25 kg ha^−1^ was applied at 20 days after transplanting (DAT).

### 2.3 Water management

Water application was done from transplanting until 60 DAT to maintain the soil moisture content at field capacity for both well-watered and 1/3 AW treatments. After 60 DAT, field capacity treatment, water was maintained at field capacity level until harvest. In the 1/3 AW treatment, irrigation was stopped at 60 DAT, and the moisture content was allowed to decrease gradually to meet the level of 1/3 AW (7.26%), which was subsequently maintained until harvest. The amount of water applied to each pot was calculated according to crop water requirement described by Doorenbos and Pruitt (1992) and surface evaporation by Singh and Russel (1981).

### 2.4 Data collection

#### 2.4.1 Soil properties and soil moisture content

Before planting, soil samples were randomly collected from 10 single samples, bulked for two samples, and examined for physicochemical properties, including soil texture (percentages of sand, silt, and clay), pH, organic matter (%), total nitrogen (N), available phosphorus (P), exchangeable potassium (K), exchangeable calcium (Ca), exchangeable magnesium (Mg), exchangeable manganese (Mn), sodium (Na), extractable iron (Fe), extractable copper (Cu), and extractable zinc (Zn) content, electrical conductivity (EC), and cation exchange capacity (CEC).

Soil moisture content (15 cm depth) was measured by the gravimetric method at 60, 75 and 90 DAT in both seasons. Briefly, soil samples were taken from each pot using a soil sampler throughout the entire column, and the soil wet weight was recorded. The soil samples were then oven-dried at 105°C for 72 h or until a constant weight was achieved, and the moisture percentage was calculated.

#### 2.4.2 Relative water content

Relative water contents of three leaflets were measured from the second fully expanded leaf between 10:00 am and 2:00 pm at 60, 75 DAT, and harvest. The leaflets were cut, immediately placed in a plastic zip bag, and stored in an ice box to prevent water loss. The leaf samples were weighted once the samples were transported to the laboratory. Then, they were placed in distilled water for 8 h, and then the turgid weight was determined. Subsequently, the leaflets were oven-dried at 80°C for 48 h or until the weight remained constant, and the leaf dry weight was recorded. Relative water content was calculated using the formula suggested by Gonzalez and Gonzalez-Vilar (2001).

#### 2.4.3 Growth, yield, and yield components

Three pots for each leaf area determination at 75 DAT, and leaf area and biomass determinations at final harvest, were separately harvested. After uprooting, the plant samples were separated into different plant parts, namely, leaves, stems, and pods. The fresh weights of each plant part were immediately recorded using an electronic balance. Leaf area was then measured using a leaf area meter (LI-3100C Area Meter, LI-COR Inc., USA). All samples were then oven-dried at 70°C for 72 h or until the weights were constant, and the dry weight was recorded. Total biomass was computed by summing the dry weights of all plant parts. Yield components were also recorded after harvesting, including number of pods per plant, pod fresh weight, pod dry weight, seed dry weight, and 100-seed weight.

#### 2.4.4 Total phenolic content and proline contents

Total phenolic content and proline content were measured in peanut leaves at 75 DAT and harvest stage. Liquid nitrogen was used to crush dry leaf samples. The total phenolic content was extracted from crushed dry leaf samples by dipping them in a beaker of pure methanol at room temperature on a magnetic stirrer. Each beaker was covered with aluminum foil. After 2 h, the extracted solutions were centrifuged at 5,000 rpm for 10 min, and the solutions were filtered through Whatman No. 4 paper. The solutions were then stored at 4°C until analysis. The total phenolic content was measured using the Folin-Ciocalteu’s reaction method (Kunnam et al., 2023), and the phenolic content was measured using a spectrophotometer (Fisher G10S UV-Vis Spectrophotometer 840-208200) at a 765-nm wavelength and expressed as gallic acid equivalent (mg of GAE 100 g^−1^ of dry samples).

Proline content was determined using the method of Bates et al. (1973). Firstly, 0.05 g of dried leaf sample was ground, and 5 ml of 3% aqueous sulfosalicylic acid solution was added into the ground sample. The solutions were then filtered through Whatman filter paper No. 1. The filtered solutions were mixed well with 2 ml glacial acetic acid and 2 ml acid ninhydrin using a vortex machine. The samples were boiled in boiling water at 98°C for 1 h, and the reaction was stopped by placing the hot samples in an ice bath. After cooling, 4 ml of toluene was added to the samples, mixed well, and set aside at room temperature to allow the solution to separate into two layers. The pink top layer of the solution was aspirated, and the absorbance was measured at 520 nm with a UV-Vis spectrophotometer.

#### 2.4.5 AMF colonization in peanut root

The colonization of AMF in plant roots was assessed according to Phillip and Hayman (1970) and Trouvelot et al. (1986) with some modifications. The roots were cleaned with distilled water before incubation at 90°C with 10% KOH for 30 min. Subsequently, the root samples were transferred to 1% HCl for 5 min and then incubated in trypan blue for 24 h at room temperature. The roots were cut into 1-cm segments, and 30 pieces were randomly sampled to assess AMF colonization under a microscope.

### 2.5 Statistical analysis

The data from all experiments were analyzed using Statistix software (version 8.0) (Statistix8, 2003). Homogeneity of variance was tested for all traits, and combined analysis of variance of two-season data was done. The means for all traits were separated by least significance difference (LSD) test at the 0.05 probability level (Gomez and Gomez, 1984). Correlation was calculated using Statistix 8 software.

## 3 Results

### 3.1 Soil chemical properties and soil moisture content

**Figure 1:**
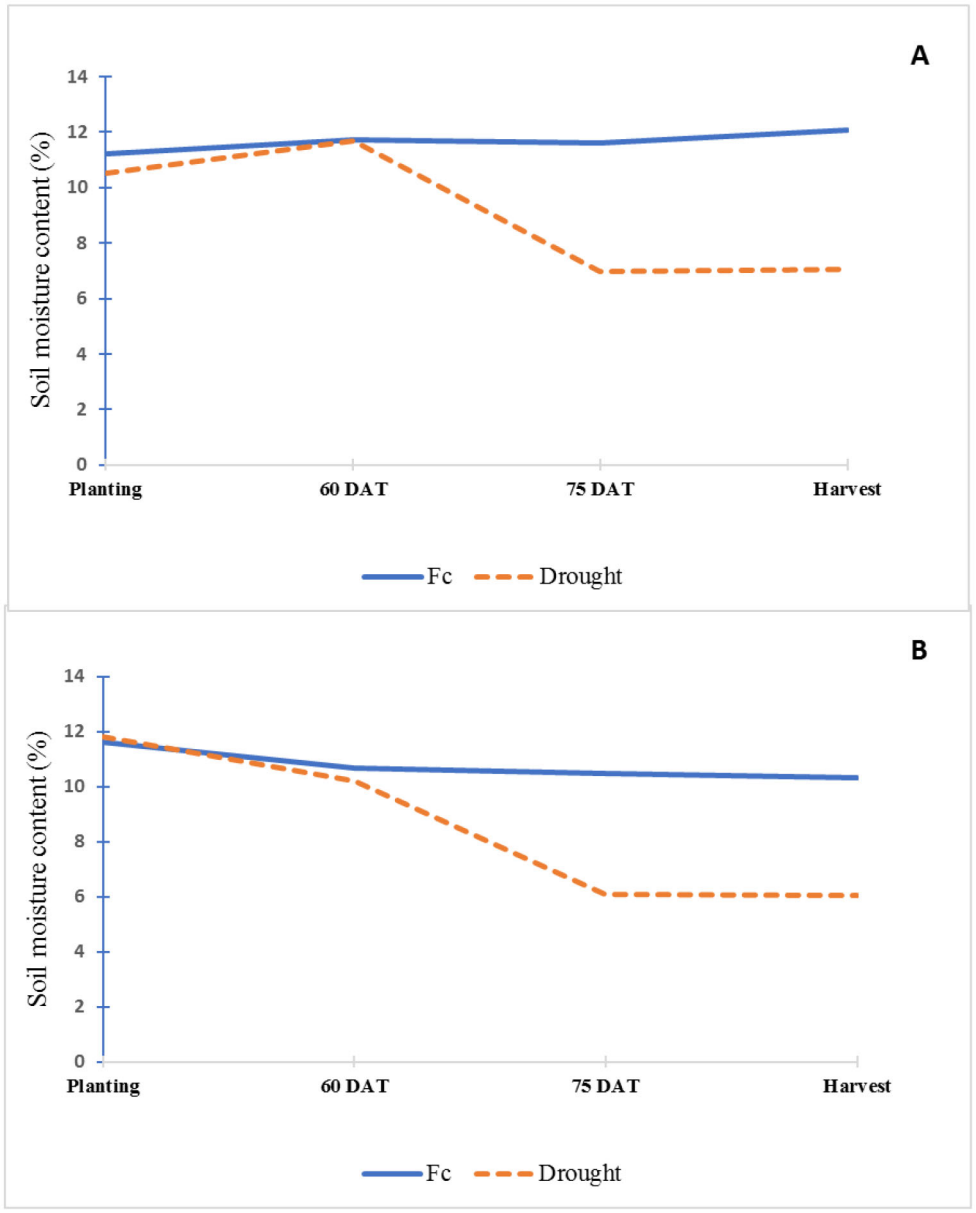
Soil moisture content (%) of field capacity (FC) and drought treatments throughout the experiment in dry **(A)** and rainy seasons **(B)**.

The soil in this study was classified as non-saline and slightly alkaline (**Table 1**). The soil was low in organic matter, exchangeable potassium, exchangeable calcium, exchangeable magnesium, extractable copper, and sodium; medium in extractable zinc; and high in available phosphorus, extractable iron, and exchangeable manganese. Soil moisture contents were significantly different between FC and drought treatment at 75 DAT and harvest stage in both seasons (**Figure 1**).

### 3.2 AMF colonization in peanut root

**Figure 2:**
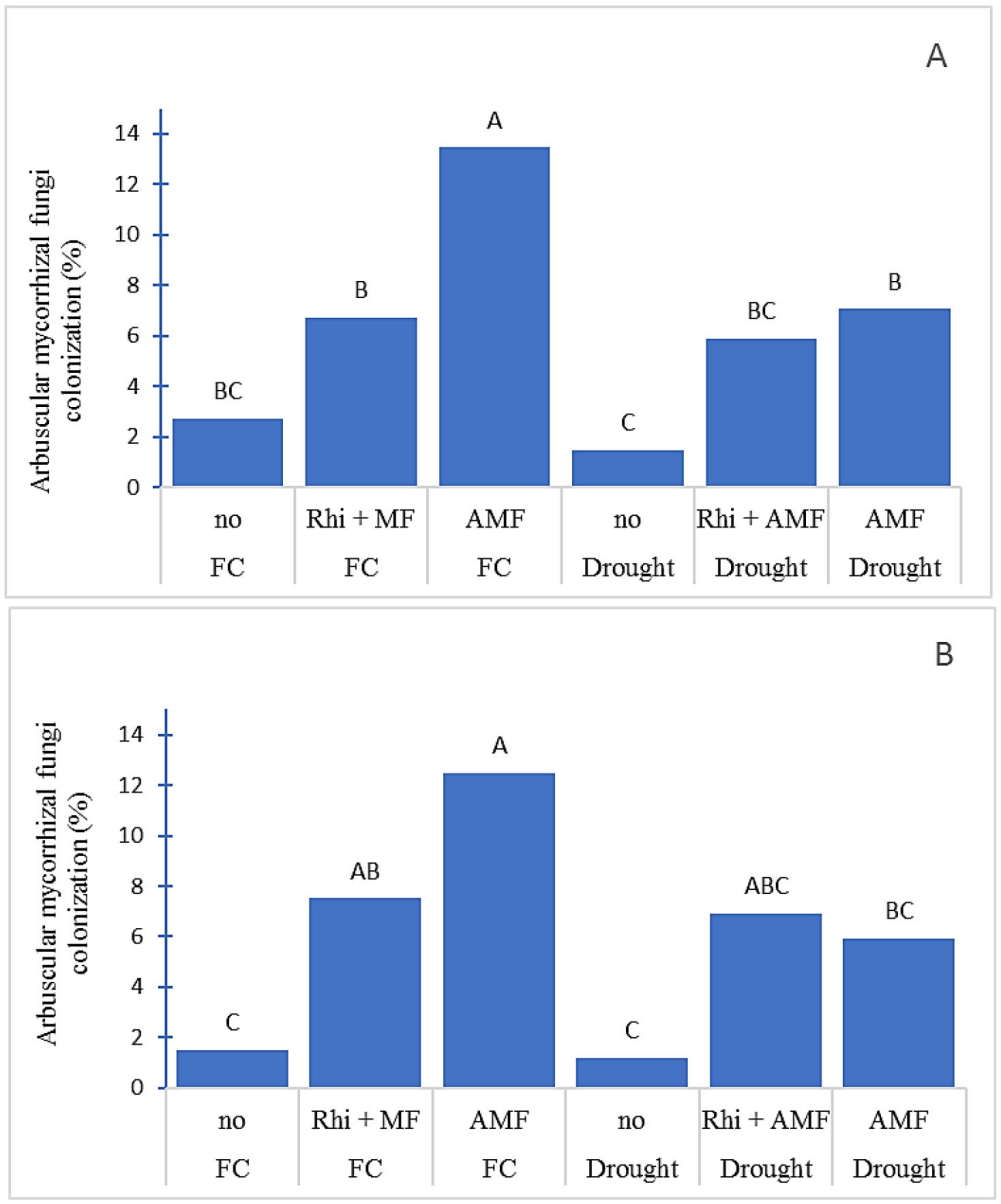
Arbuscular mycorrhizal fungi colonization (%) of peanut root growing under field capacity (FC) and drought condition in dry season **(A)** and rainy season **(B)**. no, non-inoculation; Rhi +AMF, inoculation arbuscular mycorrhiza together with rhizobium; AMF, inoculation with arbuscular mycorrhiza. The means indicated by different capital letters are significantly different at P < 0.05.

The plants grown under uninoculated treatment had lower AMF colonization than under inoculated treatments in both two water regimes and two seasons (**Figure 2**). Inoculation of AMF tended to have higher AMF colonization than co-inoculation of AMF and Rhizobium. Moreover, drought stress reduced AMF colonization in both seasons. The colonization of AMF ranged from 1.10% (uninoculated treatment under drought) to 12.48% (AMF inoculated under FC).

### 3.3 Relative water content

**Figure 3:**
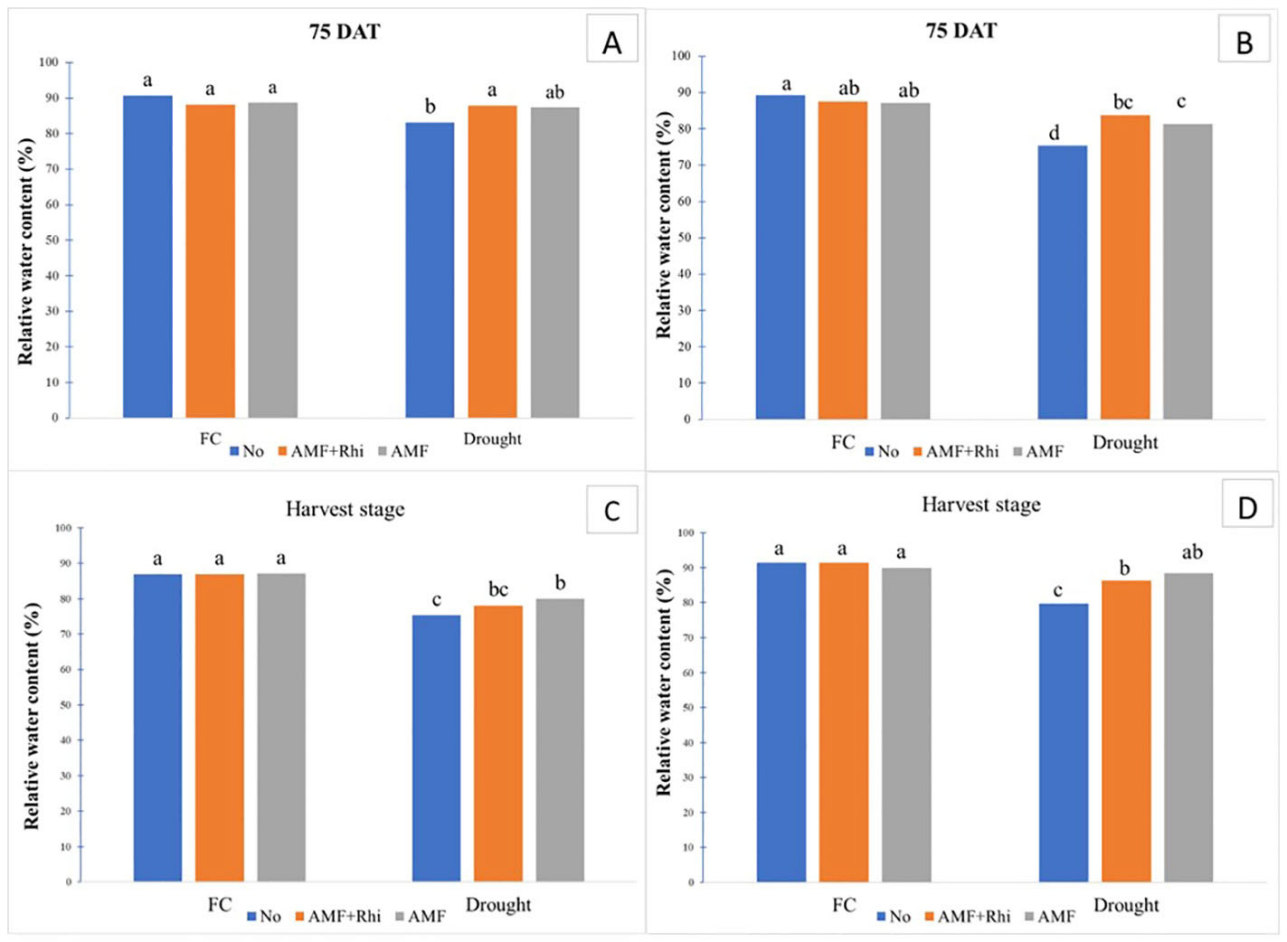
Relative water content (RWC) of peanut leaves growing under field capacity (FC) and drought condition at 75 DAT in dry season **(A)** and rainy season **(B)**, and, at harvest stage in dry season **(C)** and rainy season **(D)**. No, non-inoculation; AMF+Rhi, inoculation arbuscular mycorrhiza with rhizobium; AMF, inoculation arbuscular mycorrhiza. The means indicated by different lowercase letters are significantly different at P < 0.05.

Relative water content (RWC) decreased when plants were exposed to drought conditions in both growing seasons. The decrease in RWC was most pronounced in the rainy season (**Figure 3**).

Under FC conditions, methods of fungi inoculation did not show a significant effect on relative water content at 75 DAT and harvest in both seasons; however, they had significant effects under drought conditions. The treated plants, AMF alone and rhizobium + AMF combination, were able to improve RWC in peanut leaves under drought conditions compared with no application in both seasons, although no significant difference was observed in the dry season. Non-inoculation had the lowest RWC under drought condition.

### 3.4 Leaf area per plant

Water regime significantly affected leaf area per plant at 75 DAT and harvest in the dry season (**Table 2**). Drought stress reduced leaf area per plant from 4,650.3 cm^3^ to 4,045.6 cm^3^ at 75 DAT and 5,952.2 cm^3^ to 3,635.4 cm^3^ at harvest. The results in the rainy season were similar to those in the dry season, and drought reduced leaf area per plant from 4,739.9 cm^3^ to 3,026.9 cm^3^ at 75 DAT and from 5,826.6 cm^3^ to 3,022.3 cm^3^ at harvest.

**Table 2:** Leaf area (cm^3^ plant^−1^) per plant, leaf total phenolic content (TPC; mg 100g^−1^) and leaf proline content (mg g^−1^ dry weight) at 75 DAT and harvest stage, and biomass dry weight (g plant^−1^) at harvest stage of peanut growing under different water regimes and fungi applications in two seasons.

Treatment	75 DAT			Harvest			
	Leaf area	TPC	Proline	Leaf area	TPC	Proline	Biomass
Dry season							
Water regime							
FC	4,650.3^a^	443.55^b^	0.473^b^	5,952.2^a^	515.65^b^	0.421^b^	60.82^a^
Drought	4,045.6^b^	476.81^a^	0.590^a^	3,635.4^b^	563.18^a^	0.459^a^	39.55^b^
Fungi							
Control	4,131.6^b^	441.61^b^	0.500	4,390.9	539.49	0.447	46.20^b^
AMF+Rhi	4,471.3^a^	459.10^b^	0.508	4,687.1	533.70	0.417	52.94^a^
AMF	4,440.9^a^	479.81^a^	0.496	4,763.3	545.05	0.456	51.42^a^
Rainy season							
Water regime							
FC	4,739.9^a^	526.33^b^	0.348^b^	5,826.6^a^	510.55^b^	0.349^b^	52.43^a^
Drought	3,026.9^b^	562.98^a^	0.435^a^	3,022.3^b^	577.68^a^	0.736^a^	28.70^b^
Fungi							
Control	3,398.3^c^	517.46^b^	0.377	4,507.3	549.86^a^	0.544	38.53^b^
AMF+Rhi	4,339.3^a^	538.46^b^	0.348	4,417.4	525.50^b^	0.500	41.20^a^
AMF	3,912.5^b^	578.04^a^	0.448	4,351.6	556.98^a^	0.583	41.96^a^

Means followed by different letters in the same column in each water regimes and methods of fungi inoculation indicate significant difference (p <.05) by LSD test. Control, non-inoculation; AMF+Rhi, inoculation arbuscular mycorrhiza with rhizobium; AMF, inoculation arbuscular mycorrhiza.

Application of biofertilizers also significantly affected leaf area per plant at 75 DAT, but it did not significantly affect leaf area per plant at harvest (**Table 2**). AMF alone and AMF + rhizobium significantly increased leaf area per plant from 4,131.6 cm^3^ of control to 4,440.9 cm^3^ and 4,471.3 cm^3^, respectively. However, the difference between AMF alone and AMF + rhizobium was not significant. AMF alone and AMF + rhizobium also increased leaf area per plant at harvest, but the increases in leaf area per plant were not significant.

Inoculation of biofertilizers also significantly increased the leaf area per plant at 75 DAT in the rainy season, but the increases in leaf area per plant at harvest were not significant. AMF + Rhizobium gave the highest leaf area per plant (4,339.3 cm^3^) followed by AMF alone (3,912.5 cm^3^), whereas non-inoculated control produced the lowest leaf area per plant (3,398.3 cm^3^). However, the leaf area of the plants treated by AMF alone and AMF + rhizobium were not significantly different from un-inoculated control at harvest.

For combined analysis of the interaction between water regime and biofertilizer, it is clear that biofertilizer did not have a significant effect on leaf area per plant at the time of harvest (**Table 3**). However, biofertilizer contributed to the significantly higher leaf area per plant than untreated control at 75 DAT. The effect of biofertilizer was not significant at harvest. However, the effect of drought was more pronounced as it drastically reduced the lead leaf area per plant compared with FC.

**Table 3:** Leaf area (cm^3^) per plant, leaf total phenolic content (TPC) (mg 100g^−1^), and leaf proline content (mg g^−1^ dry weight) at 75 DAT and biomass dry weight (g plant^−1^) at harvest of peanut growing under different water regimes and fungi applications in two seasons.

Treatment	Fungi	75 DAT			Harvest			
		Leaf area	TPC	Proline	Leaf area	TPC	Proline	Biomass
Dry season								
Fc	Control	4,683.7^a^	423.10^d^	0.450^b^	6,180.6^a^	494.8^c^	0.395^d^	67.42^a^
	AMF+Rhi	4,728.1^a^	445.09^cd^	0.487^ab^	5,615.0^a^	513.8^bc^	0.407^cd^	65.75^a^
	AMF	4,539.2^ab^	462.45^bc^	0.482^ab^	6,060.9^a^	538.3^b^	0.462^ab^	64.03^a^
Drought	Control	3,579.5^d^	460.14^bc^	0.550^a^	3,681.3^b^	584.1^a^	0.500^a^	39.72^b^
	AMF+Rhi	4,214.7^c^	473.10^ab^	0.530^ab^	3,759.2^b^	556.6^ab^	0.427^bcd^	40.14^b^
	AMF	4,342.6^bc^	497.17^a^	0.510^ab^	3,465.7^b^	551.7^ab^	0.450^abc^	38.81^b^
Rainy season								
Fc	Control	4,187.3^c^	490.60^c^	0.387	5,820.2^a^	526.6^b^	0.297^c^	48.72^b^
	AMF+Rhi	5,458.0^a^	530.30^bc^	0.385	5,923.9^a^	474.8^c^	0.252^c^	55.35^a^
	AMF	4,574.3^b^	558.07^ab^	0.417	5,741.6^a^	530.1^b^	0.305^bc^	53.22^a^
Drought	Control	2,609.2^e^	544.31^b^	0.407	3,194.4^b^	573.1^a^	0.462^a^	28.35^cd^
	AMF+Rhi	3,220.6^d^	546.62^b^	0.390	2,911.0^b^	576.1^a^	0.457^a^	27.05^d^
	AMF	3,250.8^d^	598.01^a^	0.377	2,961.6^b^	583.7^a^	0.447^ab^	30.70^c^

Means followed by different letters in the same column indicate significantly different (p <.05) by LSD test. Control, non-inoculation; AMF+Rhi, inoculation arbuscular mycorrhiza with rhizobium; AMF, inoculation arbuscular mycorrhiza.

### 3.5 Total phenolic content in leaves

Drought stress was significantly higher than FC for total phenolic content in leaves of peanut at both 75 DAT and harvest (**Table 2**). Total phenolic contents of peanut grown under drought stress were 476.81 mg 100 g^−1^ at 75 DAT and 563.18 mg 100 g^−1^ at harvest, whereas total phenolic contents of peanut leaves grown under FC were 443.55 mg 100 g^−1^ at 75 DAT and 515.65 mg 100 g^−1^ at harvest. The results in the rainy season were in agreement with the results in the dry season, and total phenolic contents of peanut grown under drought were significantly higher than those of peanut grown under FC at 75 DAT and harvest.

The significantly higher total phenolic contents than untreated control was found in AMF at 75 DAT in both dry and rainy seasons. However, biofertilizer did not have significant effect on total phenolic content at harvest in the dry season, though the combined biofertilizer gave significantly lower total phenolic content (TPC) in the rainy season.

Under drought conditions, AMF-treated plants showed significantly higher total phenolic contents than untreated control at 75 DAT in both dry and rainy seasons, but the effect of biofertilizer was not significant at harvest in both seasons (**Table 3**).

### 3.6 Proline content in leaves

Drought stress significantly increased proline contents at 75 DAT and harvest in both seasons (**Table 2**). Under the dry season, the proline contents under FC were 0.473 mg g^−1^ dry weight at 75 DAT and 0.421 mg g^−1^ dry weight at harvest, whereas the proline contents under drought were 0.590 mg g^−1^ dry weight at 75 DAT and 0.459 mg g^−1^ dry weight at harvest. The effect of drought stress on proline content in the rainy season provided similar information. Biofertilizer did not have significant effect on proline content at 75 DAT and harvest in both seasons.

For interaction effect, the treatment combinations under FC were not significantly different for proline content at 75 DAT in both dry and rainy seasons (**Table 3**). However, the AMF-treated plant under FC was significantly higher than untreated control at harvest in the dry season, whereas the AMF + rhizobium-treated plant under drought stress was significantly lower than untreated control at harvest in the dry season.

### 3.7 Biomass

Fresh weight biomass and dry weight biomass provided similar information, and dry biomass was reported herein. Drought significantly reduced biomass of peanut grown in both seasons (**Table 2**). Biomass was reduced from 60.82 g plant^−1^ to 39.55 g plant^−1^ in the dry season and from 52.43 g plant^−1^ to 28.70 g plant^−1^ in the rainy season.

Application of AMF and AMF + rhizobium also significantly increased biomass in both seasons as they had significantly higher biomass than untreated control. However, the biomass of AMF and AMF + rhizobium were not significantly different.

Interaction effect showed that AMF and AMF + rhizobium were significantly higher than untreated control under FC in the rainy season (**Table 3**). However, they were not significantly different from untreated control under drought stress and FC in the dry season, and also under drought stress in the rainy season.

### 3.8 Yield and yield components

Drought stress significantly reduced all yield and yield-related traits of peanut in both seasons (**Table 4**). The reductions were from 22.89 pods to 17.21 pods for pod number, 52.23 g to 34.13 g for pod fresh weight, 26.87 g to 16.06 g for pod dry weight, 17.29 g to 9.58 g for seed dry weight, and 40.03 g to 34.27 g for 100-seed weight. Similar reductions were also found in the rainy season.

**Table 4:** Yield components of peanut growing under different water regimes and fungi applications in two seasons.

Treatment	Pod number	Pod FW (g plant^−1^)	Pod DW (g plant^−1^)	Seed DW (g plant^−1^)	100 SW (g)
Dry season					
Water regime					
FC	22.98^a^	52.23^a^	26.87^a^	17.29^a^	46.03^a^
Drought	17.31^b^	34.13^b^	16.06^b^	9.58^b^	34.27^b^
Fungi					
Control	18.08^b^	40.37	20.26	13.03	40.60
AMF+Rhi	21.58^a^	44.83	22.42	14.29	41.40
AMF	20.79^ab^	44.33	21.72	13.39	38.45
Rainy season					
Water regime					
FC	19.29^a^	37.38^a^	18.67^a^	12.35^a^	41.36^a^
Drought	12.50^b^	25.49^b^	11.07^b^	6.85^b^	34.89^b^
Fungi					
Control	15.12	33.96	15.52	9.91	35.52^b^
AMF+Rhi	17.18	30.55	14.26	8.99	38.01^ab^
AMF	15.37	31.80	14.84	9.90	40.83^a^

Means followed by different letters in the same column in each water regimes and methods of fungi inoculation indicate significantly different (p <.05) by LSD test. Control, non-inoculation; AMF+Rhi, inoculation arbuscular mycorrhiza with rhizobium; AMF, inoculation arbuscular mycorrhiza. Pod FW,Pod fresh weight; Pod DW, Pod dry weight; Seed DW, Seed dry weight; 100 SW, 100 seeds weigh.

In the dry season, the plants treated with AMF and AMF + rhizobium were not significantly different from untreated control for most traits. The significant difference was observed only for pod number in the dry season in which AMF+ Rhizobium was significantly higher than untreated control. In the rainy season, biofertilizers were not significantly different for pod number, pod fresh weight, pod dry weight, and seed dry weight, but they were significantly different for 100-seed weight. AMF was significantly higher than untreated control for this trait.

For the interaction effect, there was no significant difference between biofertilizers and untreated control for all traits under the FC and drought stress in the dry season (**Table 5**). However, in the rainy season, significant differences of biofertilizers and untreated control were observed for pod number under drought stress, pod fresh weight and pod dry weight under FC, and seed dry weight and 100-seed weight under FC and drought stress. Although there were significant differences, both biofertilizer treatments were significantly lower than untreated control under FC. In contrast to under FC, both biofertilizer treatments were significantly higher than untreated control under drought stress.

**Table 5:** Yield components of peanut growing under different water regimes and fungal applications in two seasons.

Treatment	Fungi	Pod number	Pod FW (g plant^−1^)	Pod DW (g plant^−1^)	Seed DW (g plant^−1^)	100 SW (g)
Dry season						
Fc	Control	21.16^ab^	50.70 ^a^	26.45 ^a^	17.60 ^a^	46.98 ^a^
	AMF+Rhi	24.87^a^	53.50 ^a^	27.45 ^a^	17.52 ^a^	47.64 ^a^
	AMF	22.91^a^	52.50 ^a^	26.72 ^a^	16.75 ^a^	43.47 ^ab^
Drought	Control	15.00^c^	30.05 ^b^	14.00 ^b^	8.47 ^b^	34.23 ^c^
	AMF+Rhi	18.29^bc^	36.18 ^b^	17.39 ^b^	11.07 ^b^	35.16 ^bc^
	AMF	18.66^bc^	36.16 ^b^	16.73 ^b^	10.03 ^b^	33.43 ^c^
Rainy season						
Fc	Control	19.75^a^	45.79^a^	22.43^a^	14.97^a^	44.82^a^
	AMF+Rhi	19.62^a^	32.69^b^	15.83^bc^	9.97^bc^	37.76^b^
	AMF	18.50^ab^	33.66^b^	17.70^b^	12.12^ab^	41.49^ab^
Drought	Control	10.50^d^	22.13^c^	8.61^d^	4.85^d^	26.22^c^
	AMF+Rhi	14.75^bc^	28.40^bc^	12.69^cd^	8.01^c^	38.27^b^
	AMF	12.25^cd^	25.95^bc^	11.91^cd^	7.69^cd^	40.18^ab^

Means followed by different letters in the same column indicate significantly different (*P* <.05) by LSD test. Control, non-inoculation; AMF+Rhi, inoculation arbuscular mycorrhiza with rhizobium; AMF, inoculation arbuscular mycorrhiza. Pod FW, Pod fresh weight; Pod DW, Pod dry weight; Seed DW, Seed dry weight; 100 SW, 100 seeds weigh.

### 3.9 Correlation

Water regime changed the relationships among traits of peanut (**Tables 6**, **7**), The correlation coefficients among pod number, pod fresh weight, pod dry weight, and seed dry weight were positive and significant (*P ≤* 0.01), ranging from 0.60** to 0.96** under FC and 0.89** to 0.98** under drought stress. Biomass was more important for yield under drought stress than under FC as the relationships of biomass and yield-related traits were stronger under drought stress, whereas the importance of leaf area on yield-related traits was reduced under drought stress. AMF colonization had a significant contribution to biomass under FC (0.47**); moreover, it had significant contributions to pod number (0.47*) and 100-seed weight (0.49*) under drought stress. Leaf area was more important for biomass (0.79**) and pod dry weight (0.42*) under drought stress than under FC, whereas 100-seed weight had a significant contribution to seed dry weight (0.42*) under drought stress.

**Table 6:** Correlation coefficients among AMF colonization, growth, and yield-related traits of peanut grown under FC.

	AMF colonization	Leaf area	Biomass	Pod DW	Pod FW	Pod no.	Seed DW
Leaf area	0.17						
Biomass	0.47*	−0.08					
Pod DW	−0.25	−0.02	0.26				
Pod FW	−0.27	−0.02	0.31	0.96**			
Pod no.	−0.04	−0.32	0.48*	0.70 **	0.78**		
Seed DW	−0.29	−0.04	0.09	0.94**	0.84 **	0.60**	
100 SW	−0.04	0.46*	0.15	0.35	0.28	0.02	0.33

n=24, *, **, significant at 0.05 and 0.01 probability levels, respectively.

**Table 7:** Correlation coefficients among AMF colonization, growth, and yield-related traits of peanut grown under drought stress.

	AMF colonization	Leaf area	Biomass	Pod DW	Pod FW	Pod no.	Seed DW
Leaf area	−0.19						
Biomass	−0.02	0.79**					
Pod DW	0.39	0.42*	0.55**				
Pod FW	0.38	0.37	0.49*	0.97**			
Pod no.	0.47*	0.31	0.48*	0.92**	0.93**		
Seed DW	0.41	0.38	0.48*	0.98**	0.95**	0.89**	
100 SW	0.49*	−0.16	−0.04	0.38	0.25	0.18	0.42*

n=24, *, **, significant at 0.05 and 0.01 probability levels, respectively.

## 4 Discussion

### 4.1 AMF colonization

According to the findings of this study, AMF colonization was also detected in untreated control with a small percentage of colonization. This would be due to the natural occurrence of AMF in the soil. Johnson (1998) also mentioned that the colonization of AMF in the untreated control was due to the presence of the native AMF. AMF are soil-borne microbes that play a major role in improvement of plant nutrient uptake and resistance to several abiotic stresses (Sun et al., 2018). Inoculation of effective strains of AMF can increase cop productivity under drought; therefore, the effective strains should be selected for commercial use.

In this study, single inoculation of AMF had higher colonization than co-inoculation of AMF with rhizobium. Saxena et al. (1997) stated weaker effects on AMF colonization after dual inoculation with AMF and rhizobium versus single inoculation. Chalk et al. (2006) also noted that inoculation with AMF and rhizobium resulted in reduced AMF colonization than inoculation with simply AMF and that rhizobium and AMF compete for colonization sites in legume roots, which may decrease the symbiotic effect. However, co-inoculation of AMF and rhizobium performed better than single AMF or rhizobium inoculation, and it also exhibited synergistic effects on legume microbial colonization and nodulation (Marques et al., 2001), as well as white clover growth and yield (Xie et al., 2020).

In this study, drought stress reduced AMF colonization. The reduction in AMF colonization affected by drought stress has been reported in previous research. There was no arbuscular formation and low hyphal colonization of AMF on *Populus cathayana* seedlings under extreme drought conditions. Reduction of AMF colonization may be caused by AMF death or granule formation during the drought period (Han et al., 2022).

The change in moisture regimes affected AMF colonization in root and soil (Staddon et al., 2003; Clark et al., 2009). Water content was associated with AMF colonization in *Lotus tenuis* (Garcia and Mendoza, 2008). Moisture could hasten the spores to germinate and form colonization on the roots (Yuwati et al., 2020). Higher levels of germination could be obtained at low water potential when spores were incubated longer.

The length of germ tube was reduced at low water potential (Ajeesh et al., 2015). AMF development is favored when the moisture content of the medium is slightly less than optimal for plant growth. A moisture content of approximately 0.1–0.2 bars appears to be adequate for inoculum production (Habte and Osorio, 2001).

Another factor that reduced AMF colonization is soil available phosphorus. Carrenho et al. (2007) stated that application of phosphorus significantly reduced mycorrhizal colonization in peanut. The soil used in this study had a high level of phosphorus (**Table 1**). The gene expression regulating phosphorus homeostasis in fungi was also influenced by inorganic phosphorus (Ezawa and Saito, 2018). Under high phosphorus supply, the plant can uptake sufficient phosphorus through their root; therefore, the ability of plant to limit AMF colonization is considered a strategy (Balzaegue et al., 2010; Breuillin et al., 2010). Some studies have also confirmed that the abundance of AMF can be reduced by applying phosphorus (Shao et al., 2023). The application of AMF can be effective under the appropriate water and also phosphorus concentration.

### 4.2 Relative water content

In this study, drought reduces RWC in peanut leaves, and AMF and AMF+Rhi applications can improve RWC under drought. The beneficial effect of AMF on alleviation of drought stress has been reported. Hyphae penetrate deep into the soil and provide moisture to the plants and have a positive effect on enzyme activities and protect plant cells from injury caused by drought (He et al., 2019). AMF produces an extensive mycelium which provides to absorb more nutrients and water by plant roots (Tang et al., 2022). Under drought stress, AMF also adjusted the accumulation of different hormones such as ABA, jasmonic acid (JA), and strigolactones which maintained the higher leaf relative water content and water use efficiency under drought stress. An increase in ABA level which performs as an anti-transpirant can reduce water loss by stomata closing and maintaining higher water use efficiency (Tang et al., 2022). Additionally, under stressful conditions, inoculation with AMF inhibited reactive oxygen species (ROS) accumulation of peanut; moreover, AMF-inoculated plants enhanced the activities of antioxidant enzymes (SOD, G-POD, CAT, and APX), total soluble sugar, sucrose, and free amino acids under stressful conditions (Liu et al., 2023).

In this study, AMF were higher than AMF + rhizobium for relative water content under drought stress. Under drought stress conditions, application of a mixture of bioinoculants may provide soybean plants to resist drought stress and also improve growth, productivity, and soil microbial activity (Nader et al., 2024). Ashwin et al. (2022) also stated that dual inoculation of AMF and rhizobium significantly enhanced physiological parameters and nutrient level under stress conditions. The interaction among soil microorganism is very complex and can cause the differences among studies.

### 4.3 Total phenolic content and proline content

Total phenolic content and proline content in peanut leaf were higher in drought treatment compared with well-watered conditions. Total phenolic content and proline content increased in response to drought stress. All *Achillea* species increased the proline content of the leaves, and the production of phenols in plants can be increased under drought stress (Gharibi et al., 2016). Drought stress reduces chlorophyll synthesis, increases proline contents, and causes oxidative damage by inducing the production of ROS. Under drought stress, plants accumulate different osmolytes; among them, proline is an important osmolyte that reduces the ROS by stimulating the activity of catalase (CAT), peroxidase (POD), superoxide dismutase (SOD), and other different antioxidant enzymes. Moreover, proline has a noticeable ability to bind and hydrate enzymes; thereby, it stabilizes and protects the macromolecules and maintains the structural integrity and function under drought stress (Tang et al., 2022).

In this study, AMF enhanced the total phenolic content while decreasing proline content in leaves under drought stress conditions at 75 DAT and harvest in both seasons, although there was no significant difference between AMF inoculation and non-inoculation. The proline content of AMF-inoculated plants is low, which may be due to the greater water content maintained by AMF. The finding was in agreement with previous research, which found that mycorrhiza inoculated *Cupressus atlantica* and *Erythrina variegata* and orange plants had a lower proline concentration in their leaves than non-mycorrhized plants growing under drought stress (Wu and Xia, 2006; Manoharan et al., 2010; Zarik et al., 2016).

The fungi, AMF, also improved the antioxidant defense system and the drought tolerance by increasing the accumulation of phenolic substances under drought conditions. It also considerably increases the amount of phenolic compounds by 50%–60% which substantially improves the drought stress tolerance (Tang et al., 2022).

### 4.4 Growth parameters, yield, and yield components

In this study, drought reduces leaf area, biomass, and pod yield of peanut. Fungi application can improve leaf area at 75 DAT and biomass at harvest. Drought stress takes many morphological changes in plants such as reduction in size and area of leaf and growth of root and shoot due to activation of the abscisic acid (ABA) precursor (ACC) which prevents root growth (Hewedy et al., 2021; Sayer et al., 2021). Drought stress also lessens nodule growth and their N fixation and leads to a significant reduce in the growth and production of peanut (Furlan et al., 2012). Inoculation of AMF increased leaf area, shoot dry weight, root dry weight, total dry weight, and yield of soybean (Soretire et al., 2020). The colonization of AMF increases tomato vegetative growth for 50%–60% under reduced water (Leventis et al., 2021).

However, fungi application in both methods (AMF and Rhi + AMF) did not significantly affect yield and yield components of peanut in this study. The low effects of biofertilizer in this study might be caused by low AMF colonization. The colonization of AMF in this study ranged from 1.10% (under uninoculated treatment under drought) to 12.48% (AMF inoculated under FC). The colonization of AMF in this study was low compared with co-inoculation with rhizobium in other studies. Chotangui et al. (2022) reported the AMF colonization at 50% flowering growth from 25.33% to 63.11% in peanut var. Village and Garoua.

### 4.5 Correlation

The relationships among agronomic traits and yield-related traits are well understood in peanuts. In this study, interest has been focused on the relationships between AMF colonization, and growth, yield, and yield parameters of peanut under FC and drought stress conditions.

In this study, AMF colonization could improve biomass, but it did not contribute to peanut yield under FC. However, AMF colonization could improve pod number and 100-seed weight resulting increase in seed yield under drought stress. Similar findings were reported by several former researchers. The improvement of plant performance and productivity and changes in the plant–water association can be caused by use of AMF under drought stress (Augé, 2001). Xiao et al. (2023) also indicated that AMF symbiosis proceeds drought stress tolerance of *C. migao* seedlings by improving water status, nutrient uptake, and growth. The plant under drought stress can acquire water and nutrients outside the root zone with the help of AMF (Begum et al., 2019). As a result, seed yield can be improved through the increases in pod number, 100-seed weight, and harvest index.

## 5 Conclusions

Drought reduced relative water content, leaf area, biomass, yield, and yield components in peanuts but increased proline and phenolic content in the leaves in both seasons. Application of AMF in both methods can increase relative water content, leaf area, and biomass under drought stress in both seasons; however, they had no effect on yield and yield components except pod number per plant in dry season. Fungal treatments increased phenolic levels in inoculated plants, but they did not have an effect on proline levels. It could be recommended that the use of AMF can help to preserve peanut biomass, and further research should be conducted in the fields in various growing areas.

## Data Availability

The original contributions presented in the study are included in the article/supplementary material. Further inquiries can be directed to the corresponding authors.
